# Hypothalamus-Pituitary Dysfunction as an Independent Risk Factor for Postoperative Central Nervous System Infections in Patients With Sellar Region Tumors

**DOI:** 10.3389/fendo.2021.661305

**Published:** 2021-04-30

**Authors:** Junxian Wen, Rui Yin, Yihao Chen, Jianbo Chang, Baitao Ma, Wei Zuo, Xiao Zhang, Xiaojun Ma, Ming Feng, Renzhi Wang, Wenbin Ma, Junji Wei

**Affiliations:** ^1^ Departments of Neurosurgery, Peking Union Medical College Hospital, Peking Union Medical College, Chinese Academy of Medical Sciences, Beijing, China; ^2^ Departments of Vascular Surgery, Peking Union Medical College Hospital, Peking Union Medical College, Chinese Academy of Medical Sciences, Beijing, China; ^3^ Departments of Pharmacy, Peking Union Medical College Hospital, Peking Union Medical College, Chinese Academy of Medical Sciences, Beijing, China; ^4^ Departments of Infectious Disease, Peking Union Medical College Hospital, Peking Union Medical College, Chinese Academy of Medical Sciences, Beijing, China

**Keywords:** hypothalamus-pituitary dysfunction, independent risk factor, central nervous system infections, sellar region tumors, endocrine

## Abstract

**Objective:**

The purpose of this study was to verify that hypothalamus-pituitary dysfunction is one of the risk factors for postoperative central nervous system infections (PCNSIs).

**Method:**

We performed a retrospective analysis of all patients with sellar region lesions who underwent surgery between January 2016 and November 2019 at Peking Union Medical College Hospital. In total, 44 age− and sex-matched controls were enrolled. Univariate and multivariate analyses were performed to identify risk factors for PCNSIs.

**Result:**

We enrolled 88 patients, 44 of whom had PCNSIs. Surgical approach (TCS) (P<0.001), previous surgery on the same site (P=0.001), intraoperative cerebral spinal fluid (CSF) leakage (P<0.001), postoperative adrenal insufficiency (P=0.017), postoperative DI (P=0.004) and the maximum Na^+^ levels(<0.001) correlated significantly with PCNSIs. Multivariate analysis showed that Surgery approach (TCS)(OR: 77.588; 95%CI: 7.981-754.263; P<0.001), intraoperative CSF leakage (OR: 12.906; 95%CI: 3.499-47.602; P<0.001), postoperative DI (OR: 6.999; 95%CI:1.371-35.723; P=0.019) and postoperative adrenal insufficiency (OR: 6.115; 95%CI: 1.025-36.469; P=0.047) were independent influencing factors for PCNSIs.

**Conclusion:**

TCS, intraoperative CSF leakage, postoperative DI and postoperative adrenal insufficiency are risk factors for PCNSIs in patients with sellar region tumors.

## Introduction

Central nervous system (CNS) infection is an uncommon but serious complication that can result in poor prognosis and even death. According to previous studies, the incidence of CNS infection after neurosurgical procedures is relatively variable and ranges from 0.3% to 10% (1–4). There are many causes of postoperative central nervous system infection (PCNSI), including the surgical technique, blood−brain barrier impairment, and postoperative management. Due to this urgent clinical situation, relevant predictors of PCNSI need to be identified, and prevention strategies need to be developed.

Although some studies have suggested that hypothalamus-with central nervous system infection remains unclear. A significant proportion of patients with meningitis have endocrine dysfunction (5); infection, such as sepsis, can also be caused by hypothalamus-pituitary dysfunction (6, 7). We propose that hypothalamus-pituitary dysfunction can induce PCNSI, and the purpose of this retrospective study was to test our hypothesis.

## Materials and Methods

### Study Population

A retrospective observational study of all hospitalized patients diagnosed with sellar region lesions was conducted at the Department of Neurosurgery of PUMCH. From January 2016 to November 2019, the following inclusion criteria were applied: (1) pituitary hormone deficiency alone occurring before PCNSI was classified as hypothalamus-pituitary dysfunction; (2) no preoperative infectious disease, including systemic and local infections; (3) the ability to provide informed consent; (4) clinical and radiological evidence of sellar region tumors; and (5) clinical and laboratory evidence of central nervous system infections.

Patients were required to meet at least 1 of the following criteria for the diagnosis of central nervous system infection: 1. organisms cultured from cerebral spinal fluid (CSF); 2. at least 1 sign or symptom with no other recognized cause, including fever (>38°C), headache, stiff neck, meningeal signs, cranial nerve signs, or irritability, and at least 1 criterion, including a. increased white cells (>8*10^6^), elevated protein (>0.45 g/L), and/or decreased glucose (<2.5 mmol/L) in CSF, b. organisms present based on Gram staining of CSF, c. organisms cultured from blood, d. positive antigen test using CSF, blood, or urine, e. diagnostic single-antibody titer (IgM) or 4-fold increase in paired sera (IgG) for pathogens (8).

Hypothalamus-pituitary dysfunction was defined as deficiency in one or more pituitary hormones and the presence of corresponding clinical symptoms. We collected all patients’ blood at 8AM, The diagnosis of adrenal insufficiency was based on basal dosage of cortisol below 80 nmol/L and stimulated concentrations of cortisol less than 500 nmol/L in insulin tolerance test (9). Measurement of both serum TSH and thyroxine concentrations is needed to diagnose hypothyroidism. We defined hypothyroidism base on TSH or one of the thyroxine concentrations below the reference range of the PUMCH laboratory: thyrotropin (TSH) (<0.38 µIU/mL), free T3 (fT3) (<1.8 pg/mL), free T4 (fT4) (<0.81 pg/mL), total T3 (TT3) (<0.66 ng/mL), and total T4 (TT4) (<4.3 µg/dL). The diagnostic criteria of diabetes insipidus (DI) are a 24-h urinary volume exceeding 3 L (adults) or 2 L/m2/24 h (young children), urine osmolality less than 300 mOsm/kg H2O and urine specific gravity less than 1.005 (10, 11). All patients with hypothalamus-pituitary dysfunction enrolled in this study was revised promptly and properly. For patients with adrenal insufficiency, they will oral 15mg prednisone for a week, taking 10mg at 8AM and 5mg at 4PM. In the end, the total daily dose of prednisone will gradually be reduced to 5mg based on the level of cortisol. Moreover, we treat patients with suspected adrenal crisis due to secondary adrenal insufficiency with injection of 100 mg hydrocortisone for two days, twice a day.

Each PCNSI participant was matched to a noninfection control patient, conforming to 1:1 matching for sex and age and the closest matching principle. Controls were recruited from our hospital, underwent surgery for sellar region tumors and had no symptom or laboratory evidence of PCNSI. In total, 88 patients (44 PCNSI subjects, 44 controls) were enrolled in the study. Informed consent to participate was obtained from all patients. This retrospective study was performed under the authorization of the institutional ethics committee of PUMCH, Chinese Academy of Medical Sciences.

### Data Collection

Basic information for all the patients was collected, including age, sex, and body mass index (BMI, calculated as weight in kilograms divided by the square of height in meters), signs and symptoms, white blood cell count and classification in blood and CSF, protein, glucose and chloride levels in CSF,CT and magnetic resonance imaging (MRI) examination, size of the tumor (the largest diameter of the tumor measured in the three orthogonal planes and reported by the imaging department of PUMCH), presence of underlying diseases (diabetes, hypertension), type of operative procedure, previous procedures or radiotherapy at the same location, presence of intraoperative CSF leakage, duration of hospitalization, duration of surgery, bleeding amount during the operation, presence of DI, Na^+^ levels, Prognosis and preoperative and postoperative pituitary hormones levels, including adrenocorticotropin (ACTH), cortisol, TSH, fT4, fT3, TT3, and TT4.

### Data Analysis

All data were analyzed using SPSS software version 26.0 (IBM Corp., Armonk, New York, USA). Continuous variables are described by means ± standard deviation; numbers and percentages are used for categorical variables. Univariate analyses for factors associated with central nervous system infections were performed using the chi-square test and Fisher’s exact test for categorical variables. The Wilcoxon-Mann-Whitney test and Kruskal-Wallis test were employed for continuous variables that did not fit a normal distribution. A multivariate logistic regression model was applied to select factors significantly associated with central nervous system infections. P values of 0.05 or less were considered statistically significant.

## Results

### Patient Demographics

In total, 88 patients (44 with PCNSIs, 44 controls) were enrolled in this study. The demographic information is shown in **Table 1**. The mean age of patients, including 30 men and 58 women, was 44.4 ± 14.5 ( ± SD) years old. Average BMI was 24.9 ± 4.3( ± SD) kg/m2. Most patients had undergone transsphenoidal surgery (TSS). Postoperative histological analysis revealed a large spectrum of sellar tumors, non-functioning tumors were the most common, followed by GH secretors, craniopharyngiomas and prolactinomas; with fewer numbers of cases were Rathke’s cysts, ACTH and TSH-producing adenomas.

**Table 1:** Patient characteristics and details.

Variables	n (%) or mean ± SD
Age (years)	44.4 ±14.5
BMI (kg/m2)	24.9 ± 4.3
Male	30 (34.1)
Surgery approach	
TSS	70(79.5)
TCS	18(20.5)
Pathology diagnosis	
Nonfunctioning adenomas	22 (25.0)
GH-secreting adenomas	14(15.9)
Craniopharyngioma	12(13.6)
Prolactinomas	11(12.5)
Rathke cleft cysts	10(11.4)
ACTH-producing adenomas	8(9.1)
TSH-producing adenomas	7(8.0)
Others	4 (4.5)

SD, standard deviation; TSS, transsphenoidal surgery; TCS, transcranial surgery.

The characteristics of the PCNSI patients are shown in **Table 2**. Among them, the mean WBC count increased significantly. Neutrophils accounted for the majority, followed by lymphocytes. For CSF, the WBC count of all cell counts increased, as is the level of protein. Coenocytes were the most common cell type in WBCs. There is little different of the mean glucose and chloride in the CSF of PCNSI patients compared to healthy people. In addition, the mean levels of hypothalamus-pituitary hormones and the number of postoperative DI patients were recorded. Because most patients only have one or two pituitary hormones decreased, we didn’t see a profound decrease of the mean pituitary hormone levels. There is one PCNSI patient died in our study. The direct reason of her death is Postoperative subarachnoid hemorrhage and uncontrollable Intracranial and pulmonary infections.

**Table 2:** Characteristics of PCNSI patients.

Variables	n (%) or mean ± SD
WBC (x109)	17.26 ± 7.36
neutrophil	86.99 ± 5.18
lymphocyte	8.65 ± 5.21
CSF	
Glucose (mmol/L)	3.43 ±1.69
Cl (mmol/L)	126.59 ± 8.35
Protein (g/L)	3.53 ± 2.80
Cell (X109)	48.76 ±10.85
WBC (X109)	2.61 ± 3.18
coenocyte (%)	83.59 ±11.85
monocyte (%)	16.54 ± 11.80
Hormone	
TSH (µIU/mL)	1.48 ± 1.47
TT3 (ng/mL)	0.89 ± 0.30
TT4 (µg/dL)	6.54 ± 3.19
FT3 (pg/mL)	2.48 ± 0.66
FT4 (ng/dL)	1.03 ± 0.33
Cortisol (µg/dL)	21.32 ± 34.04
DI	15(34.1)
Death	1(2.3)

SD, standard deviation; D, diabetes insipidus.

lumbar punctures were performed in all patients was suspected to have an intracranial infection. 9 patients are seen the presence of culture in CSF (**Table 3**) and 3 patients had polymicrobial infections. *Baumanii*(2/11) is common in the causative microorganisms of our study and the rest had the same frequency.

**Table 3:** The presence of culture in the CSF.

Patients	Cerebrospinal Fluid Culture	
1	*Baumanii*	
2	*Bacillus pumilus*	
3	*Enterococcus faecalis*	*Staphylococcus epidermidis*
4	*Micrococcus*	*Corynebacterium*
5	*Aerococcus aeruginosa*	
6	*Baumanii*	
7	*Pseudomonas aeruginosa*	*Streptococcus pneumoniae*
8	*Enterobacter cloacae*	
9	*Streptococcus salivarius*	

### Risk Factors for Postoperative Central Nervous System Infection

Through univariate analysis (**Table 4**), surgical approach (TCS) (P<0.001), previous surgery at the same site (P=0.001), intraoperative CSF leakage (P<0.001), postoperative adrenal insufficiency (P=0.017), postoperative DI (P=0.004) and the maximum Na^+^ levels(<0.001)correlated significantly with PCNSIs. According to multivariate analysis (**Table 5**), Surgery approach (TCS)(OR: 77.588; 95%CI: 7.981-754.263; P<0.001), intraoperative CSF leakage (OR: 12.906; 95%CI: 3.499-47.602; P<0.001), postoperative DI (OR: 6.999; 95%CI:1.371-35.723; P=0.019) and postoperative adrenal insufficiency (OR: 6.115; 95%CI: 1.025-36.469; P=0.047) were independent factors influencing PCNSI.

**Table 4:** Univariate analysis of the association between each factor and postoperative central nervous system infection.

Variable	PCNSI (n = 44)	Non-infection controls (n = 44)	P value
Age(±SD)	44.7±15.1	44.1±14.1	0.704
BMI	24.5 ± 4.2	25.2 ± 4.4	0.587
Sex			1.000
Male	15(17.0%)	15(17.0%)	
Female	29(33.0%)	29(33.0%)	
Hypertension	10(11.4%)	14(15.9%)	0.338
Diabetes	7(8.0%)	14(15.9%)	0.08
Surgery approach			<0.001
TCS	17(19.3%)	1(1.1%)	
TSS	27(30.7%)	43(48.9%)	
Previous surgery history	14(15.9%)	2(2.3%)	0.001
Previous radiotherapy	3(3.4%)	1(1.1%)	0,616*
Intraop. CSF leakage	19(27.1%)	9(12.9%)	<0.001
Postop. CSF leakage	7(15.9%)	1(2.3%)	0.058*
Postop. hypothyroidism	23(20.5%)	19(22.1%)	0.283
Postop. adrenal insufficiency	11(12.6%)	3(3.4%)	0.017
Postop. DI	15(17.0%)	4(4.5%)	0.004
Maximum Na^+^ levels	150.4 ± 7.2	143.9 ± 4.4	<0.001
Minimum Na+ levels	136.8 ± 6.0	138.2 ± 2.6	0.409

SD, standard deviation; Intraop, Intraoperative; Postop, Postoperative; *Fisher’s exact test; TSS, transsphenoidal surgery; TCS, transcranial surgery.

**Table 5:** Multivariate analysis of factors associated with postoperative central nervous system infection.

Variable	Odd ratio	95% CI	P value
Surgery approach (TCS)	77.588	7.981-754.263	<0.001
Intraop. CSF leakage	12.906	3.499-47.602	<0.001
Postop. DI	6.999	1.371-35.723	0.019
Postop. adrenal insufficiency	6.115	1.025-36.469	0.047

TCS, transcranial surgery; CI, Confidence Interval; Intraop, Intraoperative; Postop, Postoperative.

## Discussion

The sellar region is a relatively common site for brain tumors (12, 13). Over the past 30 years, significant advances in neurosurgery, neuroimaging, and molecular biology have changed the evaluation and management of sellar tumors. Nevertheless, changes in hypothalamus-pituitary function and CNS infection are still frequent postoperative complications and reasons for hospital readmissions (14–17). The incidence of hypothalamus-pituitary dysfunction is reported to be 4.2/100,000 per year, without sex differences; the prevalence is 45.5 per 100,000 people (18). The rate of PCNSIs in patients with sellar region tumors ranges from 0.5% to 14% (19). Some studies have shown the relationship between abnormal immune function and hypothalamus-pituitary dysfunction (9). Overall, it is generally recognized that patients with CNS infection might experience hypothalamus-pituitary hormone dysfunction.

In this study, we investigated pituitary hormone levels in patients with sellar region tumors and screened out those with hypothalamus-pituitary dysfunction before PCNSI. Ultimately, we found postoperative DI and postoperative adrenal insufficiency to be independent factors influencing PCNSI. Thus, we suggest that perioperative hypothalamus-pituitary dysfunction may be an underlying cause of PCNSI.

As our multivariate logistic regression analysis indicated, postoperative adrenal insufficiency significantly affected the occurrence of PCNSI (20, 21). According to a previous study, much of the excess mortality in patients with adrenal insufficiency is attributable to infectious diseases (22). Indeed, a functional hypothalamic-pituitary-adrenal (HPA) axis is essential for normal health and life expectancy. Furthermore, central adrenal insufficiency is a life-threatening disorder associated with increased morbidity and mortality (21). The possible infection-related pathogenesis pathways may involve dysregulated systemic inflammation resulting from inadequate intracellular glucocorticoid-mediated anti-inflammatory activity (23).

In addition, postoperative DI is a risk factor for PCNSI, and we speculate that the reason is impairment of plasma sodium homeostasis. Postoperative hyponatremia and hypernatremia in neurosurgical patients are typically caused by the development of DI (24). Based on inconsistencies in the definition of DI across the literature, the reported incidence of postsurgical central DI varies from 1 to 67% (25–28). The course of postoperative DI may be transient, persistent, or triphasic. In the typical triphasic response, a polyuric phase of DI is followed by an oliguric phase of SIADH and then by a third and final phase of persistent DI. When water deficits occur due to inadequate water intake to compensate for polyuria, symptoms of dehydration and/or hyperosmolality develop. Patients may present with hypernatremia in the first and third phases of DI (16, 29, 30). In addition, a hyponatremic state may result in severe metabolic derangement, myocardial depression and injury, neurologic impairment, venous thromboembolism, and poor wound healing (31). Moreover, patients with dehydration and hyperosmolality might experience a range of neurological symptoms, including irritability, cognitive decline, disorientation, and confusion, with decreased levels of consciousness, seizure and coma. Various focal neurological deficits may also develop in this context (32). In general, deterioration of the patient’s basic conditions may explain the increased risk of PCNSI that we observed.

Hyponatremia often follow after pituitary surgery. It is produced by the syndrome of SIADH and the cerebral salt-wasting syndrome (33). We don’t see the minimum Na+ levels significantly correlated to PCNSI, but Some evidence suggests that sodium is a significant promoter of immune function. Sodium acts by enhancing the function of macrophages and T lymphocytes (34, 35). A hypernatremic environment may serve as an immunological defense mechanism in inflammatory states, and sodium levels can act in concert with tissue infection as a danger signal, enhancing proinflammatory macrophage and T cell function while dampening anti-inflammatory immune responses (35). Thus, patients with hyponatremia may show decreased immune function, which may provide an explanation for the frequency of infection among patients with DI. Nevertheless, these studies generally focused on the skin and kidney, and the regulatory circuits that drive salt accumulation in the infected brain are unknown.

Postoperative hypothyroidism is not correlated significantly with PCNSI in our study. However, some evidences showed hypothalamic pituitary dysfunction could present as normal or modest increases in TSH. Moreover, in cases of chronic liver disease and chronic kidney disease patients, TSH serum concentrations could be higher than TSH biological activity (36). We didn’t measure the TSH bioactivity of patients, Consequently,it’s possible that we missed some potential patients with Postoperative hypothyroidism. The association between postoperative hypothyroidism and PCNSI need further study.

PCNSIs occurred more frequently in the TCS cohort than in the TSS cohort in our study. This result is consistent with some previous studies (3, 37). Currently, transcranial procedures are only applied in special situations, such as for a dumb bell-shaped tumor or one with irregular extensions into the frontal or temporal lobes or when TSS has failed to achieve complete tumor resection (38). This may result in an increased operation time, an increased risk of postoperative swelling or bleeding of the residual mass and the need for nonbiodegradable materials left at the completion of the TCS. These conditions may lead to an increase in the infection rate. In addition, TCS may also lead to more vigorous dissection of hypothalamus and normal pituitaries which may compromise post-operative pituitary dysfunction and bring out PCNSI.

Multivariate logistic regression analysis showed a very strong association of PCNSI development for intraoperative CSF leakage, which is already known. In fact, the risk of postoperative CSF leakage is a major impediment to the use of TSS for resection of sellar lesions, and there are clear correlations between the cranial cavity and the external environment in the event of CSF leakage. Thus, bacteria may more easily enter the cranial cavity from the external environment through the gap and cause a postoperative infection. However, skull base closure techniques have recently evolved, such that CSF leakage is no longer a significant issue following TSS.

As mentioned above, there are many causes associated with PCNSI, yet most of these factors are difficult to correct. A second operation may even be required. In contrast, hypothalamus-pituitary dysfunction can be easily identified and rectified. We believe that the results presented here will help physicians reduce the rate of PCNSI in patients with sellar region tumors.

Because of the retrospective nature of our study, our findings depend on the accuracy of the data recorded in clinical charts, which might have resulted in selection bias. It is hoped that the study findings will prompt future research.

## Conclusion

We found that TCS, postoperative DI and postoperative adrenal insufficiency are independent risk factors for PCNSI, as is intraoperative CSF leakage. Overall, awareness of hypothalamus-pituitary dysfunction may be effective to prevent PCNSIs in the future. The exact nature of the association between postoperative hypothalamus-pituitary dysfunction and PCNSI deserves further study.

## Data Availability Statement

The original contributions presented in the study are included in the article/supplementary material. Further inquiries can be directed to the corresponding author.

## Ethics Statement

This study was approved by the Ethics Committee of Peking Union Medical College Hospital (PUMCH) and written informed consent to participate was granted from all patients.

## Author Contributions

JXW and RY performed the analysis and co-wrote the manuscript. YC, JC, and BM collected the patient information. WZ, XZ, XM, and MF revised paper. RW, WM and JJW supervised the project, conceived the study, and guided the editing of the manuscript. JXW and RY contributed equally to the manuscript. All authors contributed to the article and approved the submitted version.

## Funding

This research received a grant from Beijing Tianjin Hebei basic research cooperation project [19JCZDJC64600(Z)], which support the design of the study and collection, analysis, and interpretation of data. There was no other grant from funding agencies in the public, commercial, or not-for-profit sectors.

## Conflict of Interest

The authors declare that the research was conducted in the absence of any commercial or financial relationships that could be construed as a potential conflict of interest.
